# Supplementary material for: Duration of type 2 diabetes and remission rates after bariatric surgery in Sweden 2007–2015: A registry-based cohort study
Source: PLoS Med. 2019 Nov 20;16(11):e1002985. doi: 10.1371/journal.pmed.1002985 (PMC6867594; doi:10.1371/journal.pmed.1002985)
Supplement: Attachment — Submitted filename: rebutal_plos.docx [file pmed.1002985.s002.docx]

Requests from the editors:

Title, please recast to include a study descriptor. Perhaps:

Association with bariatric surgery and the duration of type 2 diabetes in Sweden 2007-2015: a retrospective cohort study

*Authors reply: The title has been changed to include the study design (changes made to Title)*

Abstract – this is structured with 3 sections: Background, Methods and Findings and Conclusions and please ensure the final sentence of the Methods and Findings section includes a sentence on the limitations of the study.

*Authors reply: The abstract has been revised accordingly (changes made to abstract)*

Abstract – please include cities and some summary participant information such as age, sex, BMI, etc Data – an author cannot be a point of contact – please find another contact point.

*Authors reply: The abstract has been revised to include patient summary information, and the Scandinavian Obesity Surgery Registry (SOReg) has been listed as contact point. Regarding cities, the study was a nationwide study including all centers for bariatric surgery in Sweden. (abstract 43-47)*

At this stage, we ask that you include a short, non-technical Author Summary of your research to make findings accessible to a wide audience that includes both scientists and non-scientists. The Author Summary should immediately follow the Abstract in your revised manuscript. This text is subject to editorial change and should be distinct from the scientific abstract.

*Authors reply: An author summary has been added to the revised manuscript (page 3, lines 70-97)*

References in the main text – please use square brackets and remove superscript

*Authors reply: The references have been adjusted to Vancouver style accordingly (changes throughout the manuscript)*

Was written consent provided by participants?

*Authors reply: A written consent was not obtained from study participants due to the retrospective nature of the study. However, all participants have received oral and written information that baseline information and follow-up data will be recorded in the national database, and that the information will be used for research purposes. Furthermore, in accordance with Swedish legislation, all patients were given the right to deny participation. This has been clarified in the methods section of the revised version of the manuscript (methods section, page 7, lines 188-191).*

Did your study have a prospective protocol or analysis plan? Please state this (either way) early in the Methods section.

*Authors reply: No prospective protocol or analysis plan was documented or stored before the study began. However, such a plan was decided upon, and no changes were made to the original plan. The methods section has been revised to provide a more transparent description of this. (methods section page 5, lines 129-132)*

*A STROBE-reporting guidelines has been added as supplementary material. (supplementary material)*

Comments from the reviewers:

Reviewer #1: I have read with great interest the paper by Jans et al. They have used the large Swedish registry data on bariatric surgeries, to answer the question that which factors contribute to remission of T2DM after surgically-induced weight loss. The study was well-designed and -written. I have only some very minor comments to it.

Abstract

At first read, it was slightly difficult to capture the concept of correlation in the sentence: "The chance of achieving complete remission correlated…" since there were both continuous (duration of diabetes, age, Hba1c) and dichotomous variables (insulin treatment) in the analyses. Can this be opened, e.g. by adding units after each OR.

*Authors reply: Thank you for this comment! Units have been added to all ORs of continuous variables in the abstract. (changes to abstract, page 2, lines 49-50,52,54)*

Introduction

Obesity rates are low. Any data available for larger prevalences, which is the case in most countries?

*Authors reply: Originally, we only reported on the global estimation of obesity. However, we fully agree with the comments from the reviewer and have revised the introduction accordingly (Page 4, lines 104-105)*

Methods

Nro of patients in each operation type could belong to the methods, however if the authors wish, it can as well remain in the results section as it is now.

*Authors reply: Although the study is retrospective in its nature, we did not know the exact numbers of patients in each operation type when the study was planned and designed. For this reason we would prefer to keep this information in the results section. (N/A)*

Results

A flow chart of the available patients would be useful. The percentages are hard to follow. For example in the sentence "5388 patients were available for analyses on pharmaceutical use at 5 years (98%)": What does the 98% represent?

*Authors reply: Thank you for pointing this out! In accordance with the comments a Flow-chart has been added to the revised version of the manuscript. (Figure 1, Results section page 8, lines 199-201)*

Reviewer #2: Thanks for the chance to review this manuscript. This study verified that longer T2DM duration, insulin therapy prior to surgery, lower BMI were negative predictors to predict T2DM remission after RYGB/SG with a large sample size and longer follow-up period. Although these data from several registry systems was high-quality, some valuable clinical variables were missing, for instance, fasting C peptide.

Many previous studies have found T2DM duration, C peptide, age, BMI and insulin usage were powerful predictive factors, and thus the novelty of this study is limited. Additionally, during follow-up period, most patients were lost, which affected the credibility of the conclusions.

*Authors reply: Thank you for these comments! Many of the limitations were presented in the original manuscript. However, in accordance with the comments from the reviewer, we have further clarified this in the revised version of the manuscript (page 17, lines 343-344)*

Reviewer #3: Due to the imbalanced effects of bariatric surgery on clinically improving metabolic disorders among patients, exploring the factors that affect surgery-induced benefits is quite necessary. In this study, the author provides convincing evidences with large sample size to support his conclusion that the remission of T2DM induced by bariatric surgery is negatively correlated to duration of diabetes and increases in patient with recent onset diabetes and those without insulin treatment. These findings are meaningful for clinician to make decision on treating T2DM by surgery, a treatment that, although effective, cause irreversible changes in patient's GI track.

However, as the author mentioned in the manuscript, the retrospective property of this study limits its significance, other variables among subjects such as distinct behaviors during as long as 5 years may also contribute to the uneven diabetic remission rate after surgery, which need to be further and deeply explored. Whereas the high-quality data and large sample size of this study still make it convincible, and it should be eligible to be published in Plos medicine.

*Authors reply: Thank you for these comments! We have further specified examples of relevant variables not available for the study in the limitations section of the revised version of the manuscript (page 17, lines 343-344)*

Reviewer #4: I confine my remarks to statistical aspects of this paper. In general, these were fine, although I do wonder why the authors did not do a Cox proportional hazards survival analysis.

Some comments/suggestions:

Line 127-8 I think it would be better to use "proportion of excess weight". I am guessing that the people in the study varied a lot in how overweight they were. If a person is 100 kg overweight and another is 200 kg overweight and each loses 100 kg, it does not have the same effect. Maybe there is no good estimate of ideal weight, but even BMI (flawed though that is) could be used.

*Authors reply: We fully agree with the reviewer that a relative measure of weight-loss should be preferred. Both percentage total weight loss and percentage excess weight loss (or preferably excess BMI-loss) could thus be used. In accordance with the ASMBS guidelines for postoperative weight-loss (Brethauer et al, Obes Surg 2015), changes in BMI, Excess BMI-loss as well as percentage total weight loss should be used to give a complete report of postoperative weight-loss. In the original version of the manuscript no results on postoperative weight loss was presented. However, as a response to the comments from the reviewer this information is now provided within the revised version of the manuscript.*

*For the multivariable analyses (Table 3) we had to choose one method to describe weight-loss. Excess weight loss can sometimes be considered a little more complex to comprehend (for those not using this measure routinely), but in response to the comments and arguments from the reviewer, %EBMIL was used instead of %TWL. (changes to Table 3, Methods section page 6, lines 165-167, page 7 line 181, Results page 13, line 267, discussion page 16, line 323-324)*

Table 1 - What about exactly 10 years of education? or exactly3 years of higher education?

*Authors reply: Thank you for identifying this! We have clarified the length of education in the revised version of the table. (Tables 1 and 3).*

Figures - stacked histograms are not a good method (see the work of William S. Cleveland). Line charts would be better with duration on the x axis, percent on the y axis and a line for each outcome.

*Authors reply: Thank you for this valuable comment. While we agree with the reviewer on this comment from a more general perspective, it is our opinion that the stacked histograms are easily accessible and understandable for clinicians in the present manuscript. If possible, we would like to keep the histograms in the manuscript, but if this remains the strong opinion of the reviewer and the editors, we will of course provide line charts instead. (N/A)*
